# Rumor detection on social networks based on Temporal Tree Transformer

**DOI:** 10.1371/journal.pone.0320333

**Published:** 2025-04-07

**Authors:** Sirong Wu, Yuhui Deng, Junjie Liu, Xi Luo, Gengchen Sun

**Affiliations:** 1 Guangdong Provincial Key Laboratoryof Interdisciplinary Research and Application for Data Science, Beijing NormalUniversity-Hong Kong Baptist University United International College, Zhuhai,Guangdong Province, China; 2 Department of Statistics and Data Science, Faculty ofScience and Technology, Beijing Normal University-Hong Kong Baptist UniversityUnited International College, Zhuhai, Guangdong Province, China; 3 Faculty of Science,Hong Kong Baptist University, Hong Kong, China; 4 Department of PoliticalScience, Trinity College Dublin, Dublin 2, Ireland; 5 Department of Statistics andActuarial Science, Faculty of Science, The University of Hong Kong, Hong Kong,China; Kitami Institute of Technology, JAPAN

## Abstract

The rapid propagation of rumors on social media can give rise to various social issues, underscoring the necessity of swift and automated rumor detection. Existing studies typically identify rumors based on their textual or static propagation structural information, without considering the dynamic changes in the structure of rumor propagation over time. In this paper, we propose the Temporal Tree Transformer model, which simultaneously considers text, propagation structure, and temporal changes. By analyzing observing the growth of propagation tree structures in different time windows, we use Gated Recurrent Unit (GRU) to encode these trees to obtain better representations for the classification task. We evaluate our model’s performance using the PHEME dataset. In most existing studies, information leakage occurs when conversation threads from all events are randomly divided into training and test sets. We perform Leave-One-Event-Out (LOEO) cross-validation, which better reflects real-world scenarios. The experimental results show that our model achieves state-of-the-art accuracy 75.84% and Macro F1 score of 71.98%, respectively. These results demonstrate that extracting temporal features from propagation structures leads to improved model generalization.

## Introduction

Social networks have become an important medium for information dissemination and more than half of the world’s population uses social media. For hot issues that people are interest in, many content producers will try to obtain readings, reposts, and likes by taking things out of context and then exaggerating and distorting them to become popular content, which will finally become well-known to the general public and can easily form online rumors [1,2]. Since online rumors spread suddenly and quickly, they can easily cause unnecessary panic and confusion if they are not promptly screened and controlled [3,4]. Therefore, timely detection of online rumors can reduce the occurrence of unnecessary negative public affairs.

In this paper, we aim to identify the authenticity of a claim made in a conversation formed of related posts on social networks. It can be formulated into a binary classification problem, i.e. we need to identify a claim to be a rumor or non-rumor. Classical deep learning methods are usually employed to extract semantic features from post content to learn classification models. Recurrent Neural Networks (RNNs) and Convolutional Neural Networks (CNNs) are considered by Ma et al; Wu et al. [5,6] and Yu et al; Lu et al. [7,8], respectively. Generative models such as Generative Adversarial Networks (GANs) and Variational Autoencoders (VAEs) are also used to capture the latent representation of a rumor [1,9,10]. To solve the problem of short-term memory, Transformer-based models are used to extract the semantic features among different words by self-attention mechanism [11–13]. However, classification models based solely on semantic features often do not have good generalization ability due to the fact that certain types of rumor events are more likely to show their unique textual features. Thus it is necessary to utilize more features of rumors to learn a supervised classifier.

Rumors on social networks have the characteristic of spreading as a social contagion since online media users like sharing their opinions, conjectures, and evidence of inaccurate information with others [14,15]. Thus, the propagation patterns of rumors generated by interactions among posts can help us identify the authenticity of a claim made in an event. Posts and their interactions are usually modeled as nodes and edges in graphs. The current research study on the propagation characteristics of rumors can be categorized into two groups.

One line of work utilizes the tree structure, a directed acyclic graph, to model propagation structure. Shang et al. [16] pointed out that the information propagation in social networks exhibits a tree-like structure. A tree-structured model can effectively and intuitively represent the changes in the direction of rumor propagation and the evolution of semantics. Kumar and Carley [17] used binarized constituency trees to compare features in source posts and their replies. Ma et al. [18] used recursive neural networks with a tree structure to learn the structure of rumor spread. Ahmed et al. [19] proposed Tree Transformer for sentence representations by performing recursive traversal only with attention. Ma and Gao [11] further improved the representation power of the Tree Transformer [19] by adding a post-level self-attention module and achieved better rumor detection performance.

Another line of work introduces Graph Neural Networks (GNNs) to model the complex topological structures involved in rumor propagation [8,20–22]. In the spread of a rumor, some comments may question and present evidence contradicting the original post, leading to repeated semantic evolution through these contradictions. Ye et al. [23] combines rumor content and propagation structure information based on a graph model to explore their interactions during the rumor propagation process, thereby obtaining a representation of the rumor. Although GNNs are adept at capturing global information and effectively characterizing the features of nodes and edges, they inherently treat all nodes as existing on the same hierarchical level. This uniform approach overlooks the dynamic and nuanced semantic evolution that occurs during rumor propagation. As a result, GNNs have a limitation in their ability to account for and adapt to the continuous changes in semantics introduced by the interplay of supporting and contradicting information. Tao et al. [24] enhanced their approach by integrating the encoding of parent-child node pairs with GNNs to capture the semantic changes between tweets and their responses more effectively.

**Fig 1:**
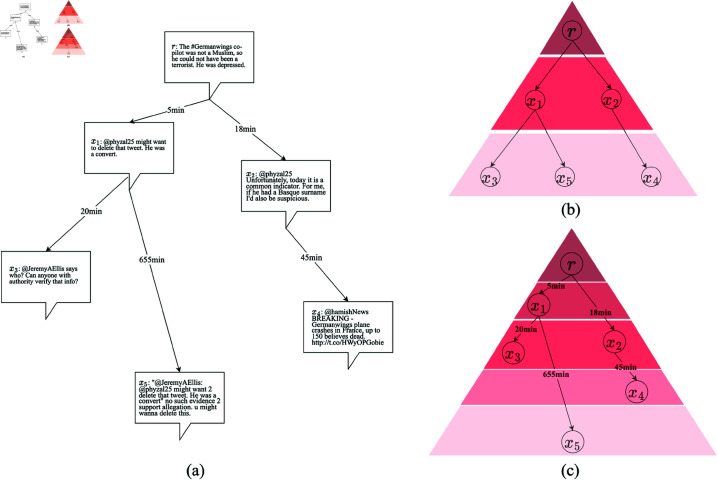
An example from PHEME dataset collected from Twitter by Zubiaga et al. [27]. A claim labeled as a rumor about Germanwings crash event. (a) The real conversation thread with spatial and temporal information. (b) The spatio-only propagation tree structure which is obtained by hierarchically aggregated by the root post *r*’s first generation {*x*_1_,*x*_2_} and second generation {*x*_3_,*x*_4_,*x*_5_}. (c) The spatio-temporal propagation tree structure with five hierarchies generated from (a). It allows us to observe finer-grained propagation structure with both spatial and temporal information, and then to better extract the propagation patterns of rumors.

In the above graph structure models, nodes from the same generation are hierarchically aggregated to generate an entire conversation thread. However, this spatio-only propagation structure does not capture the growth of a real conversation thread along the time. Thus it is necessary to consider temporal information which can help us to observe finer-grained propagation structures, and thus to extract more information in propagation patterns of rumors. Yu et al. [25] divided the cascade into sub-cascade graphs based on temporal development and used a graph-based network to learn their local structural information. Their research showed that extracting dynamic features is crucial for predicting cascade size. Huang et al. [26] also claimed that the propagation tree structure can be further differentiated by their temporal structures since it can reveal differences in the propagation path of information. For instance, Fig 1 shows an example from PHEME dataset collected from Twitter by Zubiaga et al. [27]. The claim is labeled as a rumor about the crash of a Germanwings plane. Fig 1a shows the real conversation thread with spatial and temporal information. For simplicity, the root post and other posts are denoted as  { *r* }  and {*x*_1_,*x*_2_*x*_3_,*x*_4_,*x*_5_}, respectively. Fig 1b shows the spatio-only propagation tree structure which is obtained by hierarchical aggregating the root post *r*’s first generation {*x*_1_,*x*_2_} and second generation {*x*_3_,*x*_4_,*x*_5_}. In this structure, *x*_3_ and *x*_5_ are in the same hierarchy. However, as shown in Fig 1a, *x*_3_ replies *x*_1_ in twenty minutes while *x*_5_ replies it after almost eleven hours during which the user is exposed to more external information. Thus the information provided by these two propagation nodes is different for detection of rumors. Fig 1c shows a spatio-temporal propagation tree structure with five hierarchies generated from the original conversation thread. It allows us to observe finer-grained propagation structure with both spatial and temporal information, and then to better extract the propagation patterns of rumors.

Although semantic features in posts, spatial and temporal features in propagation structures are significant for us to detect rumors on social networks, few researches consider these three features simultaneously [5,11,21,24,28,29]. In this paper, we propose a composite architecture Temporal Tree Transformer model which can simultaneously extract semantic, spatial and temporal propagation features to detect rumors. Equal-depth time window is first applied to observe the growth of propagation trees from subtrees to an entire tree. Then the Tree Transformer model proposed by Ma and Gao [11] is used to hierarchically encode the subtrees in different time window. Since Gated Recurrent Unit (GRU) demonstrates greater advantages in extracting temporal information, particularly in tasks involving long sequences, limited data, strong local dependencies, or noisy time-series data [30–32], we employ GRU on all subtrees to extract features of rumors. To avoid information leakage caused by randomly splitting conversation threads from all events into training and test sets, and to evaluate the performance of our model in a more realistic scenario, we adopt the Leave-One-Event-Out (LOEO) principle. That is, one event is used as a test set and the remaining events are used as a training set in each iteration. LOEO principle constructs a test environment more close to real-world scenarios and better evaluates the ability of model generalization.

The contributions of this work are as follows:

- We characterize post propagation from a more fine-grained perspective, i.e., temporal features as well as spatial features are extracted from propagation structures to achieve high performance of our classification model.
- Rather than randomly slicing the conversation threads from all events into training and test sets, a more realistic principle, Leave-One-Event-Out (LOEO), is used for our validation. It is more suitable for practical application scenarios, and thus it better evaluates the ability of model generalization.

The rest of this paper is organized as follows. We define our problem statement in Section Probelm Statement and introduce the proposed model in Section Proposed Model. In Section Experiments and Results, we describe the dataset and baselines we use in our experiments and show our experimental results. We then conclude with future work in Section Conclusions and Future Discussion.

## Problem statement

We aim to predict the authenticity of a claim made in a conversation given its source post, response posts and their interactions. Let *V*(*r*)={*r*,*x*_1_,*x*_2_,…,*x_n_*} denote the conversation thread of an event, where *r* is the source post, *x_i_* is the *i*-th response post in chronological order, and *n* is the number of response posts in the thread. To capture the spatial and temporal features in thread propagation, we use tree structure to model the propagation, and observe the growth of the trees from different time windows. Since time interval between a post and its response has large variance in different conversation threads, using time window with equal width to observe the growth is not practical. Thus equal-depth time window is used, that is, there are same number of posts in each time window. Let *k* denote the number of posts in each time window, then a conversation thread with a root post *r* and *n* response posts can be divided into $\left\lceil \frac{n+1}{k}\right\rceil$ time windows in chronological order, where  ⌈ ⋅ ⌉  denotes the integer obtained from rounding up. The posts from the first time window form an initial subtree, and then the posts from the second time window are aggregated to the initial subtree to form the second subtree and so forth. The aggregation stops when an entire propagation tree is formed. For instance, in the conversation thread $V(r)=\{r,x_{1},x_{2},\ldots ,x_{5}\}$ shown in Fig 1a, we observe the growth of the propagation tree through three subtrees {*r*,*x*_1_, {*r*,*x*_1_,*x*_2_,*x*_3_} and {*r*,*x*_1_,*x*_2_,*x*_3_,*x*_4_,*x*_5_} from three different time windows if *k* = 2 is applied to divide the thread. We propose Temporal Tree Transformer model to extract semantic, spatial and temporal features from these propagation subtree structures, and thus to detect rumors on social networks.

## Proposed model

**Fig 2:**
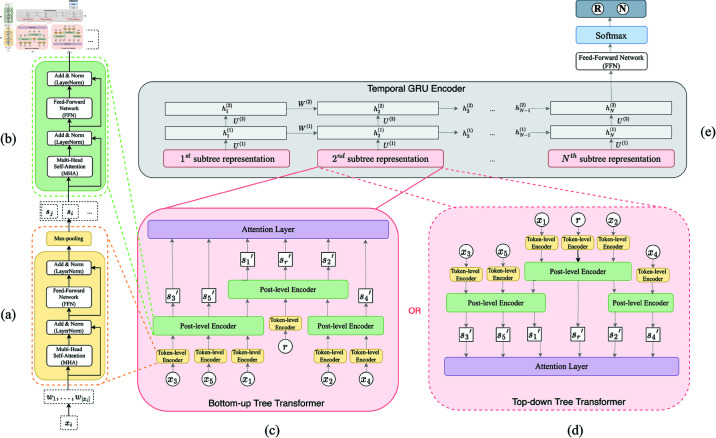
Temporal Tree Transformer. (a) Token-level Transformer Encoder. (b) Post-level Transformer Encoder. (c) and (d) are Tree Transformer with Bottom-up and Top-down integration, respectively. (e) Temporal GRU Encoder.

Temporal Tree Transformer consists of three components: (1)Token-level Transformer Encoder: all posts in a conversation thread are encoded using Token-level Transformer Encoder; (2) Tree Transformer: All posts in a subtree formed in a given time window are hierarchically encoded using the Post-level Transformer Encoder on a continuous basis. It enhances the representation of some posts in the subtree. One of two ways: Bottom-up or Top-down is employed to integrate information and then a subtree representation is obtained; (3) Temporal GRU Encoder: all subtrees in the conversation thread are encoded using a GRU. Finally a Softmax funciton is employed to obtain a probability of being a rumor for the conversation thread. Fig 2 gives an overview of Temporal Tree Transformer, the details of which will be explained in the folowing subsections.

### Token-level Transformer Encoder

All nonalphabetic characters and stop words are removed from each post, and the rest words are converted to lowercase. BERT-Tokenizer^1^ is used to obtain the initial token embedding for each word in a post [33]. Then the *i*-th post *x_i_* can be initially represented as $x_{i}=(w_{1},w_{2},\ldots ,w_{t},\ldots ,w_{\left|x_{i}\right|})$, where *w_t_* is a *d*-dimensional embedding vector of the *t*-th word and |*x_i_*| denotes the number of words in *x_i_*. As shown in Fig 2a, Multi-Head Attention (MHA) is applied to *x_i_*, then the output $x_{i}^{MHA}$ is fed to two normalization sublayers (LayerNorm) and a fully connected feed-forward sublayer (FFN). Finally max-pooling is applied to the output matrix to obtain $s_{i}\in {\mathbb{R}}^{d\times 1}$ which is the vector representation for post *x_i_*. It can be formulated as,


$$\begin{array}{ccc} \begin{array}{ccc} L_{i}^{MHA} & =LayerNorm(x_{i}^{MHA}+x_{i}) & \\ H_{i} & =FFN(L_{i}^{MHA})\\ L_{i}^{FFN} & =LayerNorm(L_{i}^{MHA}+H_{i})\\ s_{i} & =MaxPooling(L_{i}^{FFN}) \end{array} & & \end{array}$$ (1)

### Tree Transformer

Within a given time window, all the posts in the propagation subtree are encoded by Tree Transformer proposed by Ma and Gao [11]. It hierarchically encodes and integrates posts using Post-level Transformer Encoder on a continuous basis. As shown in Fig 2b, Post-level Transformer Encoder is similar to Token-level Transformer Encoder but without max-pooling layer. One of two ways, Bottom-up and Top-down is applied to hierarchically integrate the encoded representations containing semantic and propagation information. In the Bottom-up way, let *x_j_* denote one of the deepest parent nodes in the subtree, and *s_j_* is its post-level representation. For every *s_j_*, a Post-level Transformer Encoder is used to encode *s_j_* and its child notes. Since the deepest parent nodes are also child notes of other higher level parent nodes, their first representations as well as the token-level representations of their parents are encoded again using Post-level Transformer Encoder. The above encoding process is continuously implemented until the root node is reached, and thus the post-level representation for an entire subtree in a given time window, $S^{\prime }(r)=\{s_{r}^{\prime },s_{1}^{\prime },s_{2}^{\prime },\ldots \}$ where *r* is the source post, is obtained. For instance, Fig 2c shows a Bottom-up way of integration on the tree structure given in Fig 1b. *x*_1_ and *x*_2_ are two deepest parent nodes in the tree, and their token-level representations are *s*_1_ and *s*_2_. Two subtrees {*s*_1_,*s*_2_,*s*_3_} and {*s*_2_,*s*_4_} are first encoded through Post-level Transformer Encoder, respectively. The parent nodes *s*_1_ and *s*_2_ are also child nodes in the higher level subtree {*r*,*s*_1_,*s*_2_}, thus their first representations together with the token-level representation of their parent node *r* are encoded again through Post-level Transformer Encoder. Finally the post-level representation for the entire tree, i.e. $S^{\prime }(r)=\{s_{r}^{\prime },s_{1}^{\prime },s_{2}^{\prime },s_{3}^{\prime },s_{4}^{\prime },s_{5}^{\prime }\}$ is obtained. Similarly, Top-down integration integrates information from top to bottom. For instance, as it is shown in Fig 2d, the top subtree {*r*,*s*_1_,*s*_2_} is first encoded. Then its child nodes *s*_1_ and *s*_2_ are encoded again in lower level subtrees {*s*_1_,*s*_2_,*s*_3_} and {*s*_2_,*s*_4_} due to the fact that they are parent nodes in these two subtrees. Finally the post level representation for the entire tree *S′*(*r*) can be also obtained. The way of Top-down integration is more similar to the nature of information propagation in social networks.

After all nodes are encoded by Tree Transformer, some of them carry significant information of claims and stances stated for the entire subtree. Then an attention layer is employed to weight each node and thus the representation for the entire subtree $\tilde{s}$ can be written as a weighted sum of post-level representations for all nodes which contain both semantic and structure information.


$$\begin{array}{ccc} \tilde{s}=\underset{i}{\sum }\alpha_{i}\cdot {s_{i}}^{\prime },\;\alpha_{i}=\frac{exp(\mu^{⊤}{s_{i}}^{\prime })}{\underset{j}{\sum }exp(\mu^{⊤}{s_{j}}^{\prime })} & & \end{array}$$ (2)

where ${s_{i}}^{\prime }$ is the post-level representation of the *i*-th node, $\mu \in {\mathbb{R}}^{d\times 1}$ is the transformation weight. $\alpha_{i}\in [0,1]$ is the attention weight of ${s_{i}}^{\prime }$ obtained from applying softmax function on $\mu^{⊤}{s_{i}}^{\prime }$. It measures the importance of information carried by different nodes.

### Temporal GRU Encoder

To extract temporal features in propagation, we observe the growth of propagation trees from different time windows. Since the text and structural feature extraction methods we employed are based on Transformer models, it might seem natural to also use a Transformer model for extracting temporal features. However, as Lim B. et al. [31] highlighted, Gated Recurrent Unit (GRU) outperforms Transformer in scenarios involving small-scale datasets and long time-series tasks due to its simpler structure and more precise modeling of local temporal dependencies. Therefore, we opted to use GRU for temporal feature extraction. Let ${\tilde{s}}_{t}$ denote the representation for the subtree from the *t*-th time window using the above Tree Transformer. Then all these representations can be encoded using GRU, as shown in Fig 2e to extract temporal features. The GRU layer [34] is formulated as,


$$\begin{array}{ccc} \begin{array}{ccc} z_{t} & =\sigma ({\tilde{s}}_{t}U_{z}+h_{t-1}W_{z}) & \\ r_{t} & =\sigma ({\tilde{s}}_{t}U_{r}+h_{t-1}W_{r})\\ {\tilde{h}}_{t} & =tanh({\tilde{s}}_{t}U_{h}+(h_{t-1}\cdot r_{t})W_{h})\\ h_{t} & =(1-z_{t})h_{t-1}+z_{t}{\tilde{h}}_{t} \end{array} & & \end{array}$$ (3)

where *U_z_*, *W_z_*, *U_r_*, *W_r_*, *U_h_*, and *W_h_* are weight matrices to be learned in GRU. The output is the hidden vector *h_N_* of the last GRU unit, where $N=\lceil \frac{n+1}{k}\rceil$ is the number of time windows. Then we use a fully connected layer and Softmax function to obtain a probability of being a rumor for the conversation thread, and thus to obtain the binary classification output *ŷ* ∈ { Rumor, Non-rumor } . In our experiments, we also compare the performance of Transformer and GRU for temporal feature extraction. As presented in Table 2, the results demonstrate that GRU achieved superior performance.

### Model loss

The model loss is written as summation of categorical cross-entropy loss (CrossEntropy) and *L*_2_ regularization term, which is formulated as


$$\begin{array}{ccc} L(y,ŷ)=\overset{N}{\underset{i=1}{\sum }}CrossEntropy(y_{i},\hat{y_{i}})+\lambda \cdot {∥\Theta ∥}_{2}^{2} & & \end{array}$$ (4)

where ${ŷ}_{i}$ denotes the predicted class of *i*-th training data and *y_i_* denotes the corresponding true label, ${∥\cdot ∥}_{2}^{2}$ denotes the *L*_2_ regularization term over all the model parameters *Θ* and *λ* denotes the trade-off coefficient.

## Experiments and results

### Dataset

We evaluate our model on the public dataset PHEME collected from Twitter by Zubiaga et al. [27]. It is the only dataset which contains both the propagation path of rumor and response posts. The PHEME dataset contains five breaking events including shooting at Charlie Hebdo (CH), the hostage situation in Sydney (SS), Ferguson unrest (FG), shooting in Ottawa shooting (OS), and the crash of a Germanwings plane (GC). Each thread in PHEME dataset is labeled with two classes, rumor and non-rumor. Events differ in size drastically and have different class-label proportions which illustrate unbalanced distributions for rumors. Table 1 shows the basic statistics of PHEME dataset.

**Table 1:** Statistics of PHEME v5 dataset.

Statistics	CH	SS	FG	OS	GC	Total
Threads	2,002	1,173	1,010	857	405	5,447
Posts	39,286	24,511	25,673	12,735	5,178	107,383
Non-rumors	1,555	673	753	400	203	3584
Rumors	447	500	257	457	202	1863
Average tree depths	3.82	4.06	4.06	2.84	2.51	3.67
Max tree depths	22	19	47	19	19	47
Average tree posts	19.62	20.9	25.42	14.86	12.79	19.71
Max tree posts	347	343	297	111	82	347

### Experimental setup

**Fig 3:**
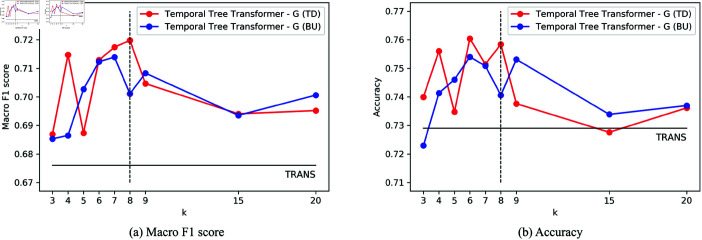
The effects on Macro F1 score and accuracy of varying the value of *k* in LOEO validation for TTT - G. The red lines represent Top-down, while the blue lines represent Bottom-up. In both figures, the TRANS results are marked as black lines.

In the training process, parameters in the Temporal Tree Transformer are updated by backpropagation with Adaptive Moment Estimation Algorithm (Adam) optimizer. We set the word embeded dimension *d* to be 768 and the hidden dimension for fully connected layer to be 600. We apply one layer of Transformer to token-level encoder while six layers of Transformer to post-level encoder, and the head number for both MHA modules is 12. During training, the learning rate is set as 0.000042 and the dropout rate is set as 0.2. The time window depth *k*, which denotes the number of posts in each observing time window, is set to 8 according to the experimental results of *k* as shown in Fig 3.

### Models for comparison

To show the effectiveness of our proposed model, we compare the results with the following models on rumor detection classification problems.

- RNN: A RNN-based model [5] is used to learn semantic information with sequential structure.
- CNN: A CNN-based model [7] is used to extract key semantic features and their interactions from conversation sequences.
- MTL3: A multi-task learning model is used for joint tasks for rumor detection, rumor tracking, and stance classification [35].
- GAN-RNN: Generative model GAN is used with RNN to enhance semantic representation [29].
- GACL: A GNN-based model with contrastive learning [21] that considers the propagation structure information of rumors.
- GARD: The latest rumor detection model [24] that integrates the encoding of parent-child node pairs with GNNs to effectively capture both local semantic changes and global structural information.
- TRANS: A Tree Transformer model [11] considers both semantic and propagation information based on tree structures.
- TTT - T, TTT - G: Our proposed Temporal Tree Transformer models with Transformer and GRU in temporal encoders, respectively.

### Rumor classification performance

Five events in PHEME dataset suffer from the class imbalance problem. When faced with data skewness or class imbalance, a classification task is significantly hindered. In such situations, relying solely on accuracy score as an evaluation metric is insufficient due to its limitations in effectively handling class imbalance problems. Thus, to accurately capture the performance of models on PHEME dataset, we refer to the evaluation framework in Zhang et al. [36] which includes accuracy, precision, recall, Macro F1 score, and AUC. Rather than randomly slicing the conversation threads from all five events into training and test sets, a more realistic principle, Leave-One-Event-Out (LOEO), is used for our validation. It is more suitable for practical application scenarios, and thus it better evaluates the ability of model generalization.

The results of our experiments are presented in Table 2. It lists a comparison between our proposed Temporal Tree Transformer model (TTT) with other baseline models. T and G indicate Transformer and GRU in temporal encoders, respectively. BU and TD indicate Bottom-up and Top-down, respectively. The notation ‘-’ indicates that the original paper did not give the relevant results. It shows that our Temporal Tree Transformer models achieve higher accuracy and Macro F1 scores than other models. Among all the baselines, the TRANS model performs best as it fully utilizes semantic and spatial propagation information with strong representation power of the Transformer model. It is important to highlight that GACL and GARD are models grounded in GNNs. These models leverage a robust GNN encoder to capture the global structural features of rumor propagation. However, the tree-structured model TRANS demonstrates superior generalization ability in LOEO validation scenarios compared to GNN-based models. Among our Temporal Tree Transformer models, TTT - G (TD) achieves the best performance due to the fact that GRU Encoder with lower complexity is more suitable for rumor time series data and the way of TD integration is more similar to the nature of information propagation in social networks. TTT - G (TD) model makes a significant improvement over the TRANS model by 2.18%–2.94% (3.74%–4.38%) in terms of accuracy (Macro F1 score).

**Table 2:** Experimental results on PHEME dataset.

Method	Accuracy	Macro F1	Rumor F1	Non-rumor F1	Precision	Recall	AUC
RNN	0.7142	0.6733	0.5728	0.7738	0.5660	0.5798	0.6743
CNN	0.6406	0.5994	—	—	—	—	—
MTL3	0.6850	0.6400	0.5640	0.7160	0.4978	0.6504	0.6547
GAN-RNN	0.6866	0.6666	—	—	—	—	—
GACL	0.7079	0.6665	0.5663	0.7667	0.5540	0.5791	0.6684
GARD	0.7273	0.6822	0.5858	0.7786	0.5754	0.5966	0.6839
TRANS	0.7290	0.6760	0.5585	0.7934	0.6024	0.5205	0.6710
TTT - T (BU)	0.7460	0.7027	0.6067	0.7987	0.6130	0.6005	0.7017
TTT - T (TD)	0.7514	0.7174	0.6397	0.7952	0.6778	0.6056	0.7242
TTT - G (BU)	0.7508	0.7134	0.6350	0.7928	0.6817	0.6089	0.7185
TTT - G (TD)	0.7584	0.7198	0.6405	0.7991	**0.7028**	**0.6101**	**0.7240**

Table 3 shows the details of our model performance for each unseen event. The ‘Event’ column shows five different events used as a test set in LOEO validation. The model has the best results on the test event Charlie Hebdo (CH). The reason is that the training set has reasonable proportion of two classes.

**Table 3:** Experimental results of Temporal Tree Transformer in each event.

Method	Event	Accuracy	Macro F1	Rumor F1	Non-rumor F1	Precision	Recall	AUC
TTT - G (BU)	CH	0.8307	0.7742	0.6613	0.8871	0.5974	0.7404	0.7985
	SS	0.7329	0.7223	0.6681	0.7766	0.7104	0.6306	0.7198
	FG	0.7614	0.6470	0.4460	0.8479	0.5447	0.3776	0.6349
	OS	0.7270	0.724	0.6953	0.7526	0.8584	0.5843	0.7371
	GC	0.7022	0.7022	0.7044	0.7000	0.6976	0.7114	0.7022
TTT - G (TD)	CH	0.8415	0.7825	0.6694	0.8955	0.6254	0.7201	0.7980
	SS	0.7483	0.7433	0.7076	0.7790	0.7008	0.7146	0.7439
	FG	0.7752	0.6533	0.4477	0.8589	0.5973	0.3580	0.6378
	OS	0.7025	0.6954	0.6492	0.7416	0.8740	0.5164	0.7157
	GC	0.7246	0.7245	0.7286	0.7204	0.7164	0.7413	0.7246

We further investigate the effects of the hyperparameter *k* which denotes the number of posts in each time window. The specific experimental results are provided in the tables in S1 Table and S2 Table. To intuitively determine the most suitable value for hyperparameter *k*, we have plotted a line chart, as shown in Fig 3. It is observed that with *k* set to 8, our model TTT - G (TD) achieves the highest Mac F1 score and relatively high accuracy in LOEO validation. Even when the value of *k* increases to 20, our model is robust and shows better performance than the TRANS model. As the assessment criteria is task-oriented, therefore, we set the hyperparameter *k* of our framework to 8 to compare with other models.

**Fig 4:**
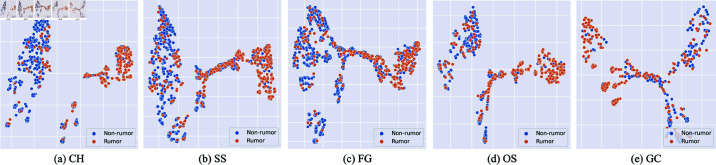
Visualization of the latent representation for conversation threads in five events using t-SNE. Orange dots represent Rumor class, while blue dots represent Non-rumor class. The two clusters are separated in all five subplots, demonstrating the well prediction ability of our classification model in unseen events.

To examine the effectiveness of our conversation representations for classification task, we use t-SNE proposed by Van der Maaten and Hinton [37] to visualize the separability of representations obtained from TTT - G (TD). Fig 4 shows the clusters of points obtained from t-SNE. Orange and blue dots denote the Rumor and Non-rumor classes predicted by our model, respectively. Two clusters are distinct from each other in five events. This further shows that our Temporal Tree Transformer model generates good representations for rumor detection in unseen events.

## Conclusions and future discussion

In this paper, we aim to identify the authenticity of a claim made in a conversation thread formed of related posts on social networks. To achieve this, we use equal-depth time window to observe the growth of propagation tree structures. In each time window, the tree is encoded using Token-level Transformer Encoder and Tree Transformer in one of two ways, Bottom-up or Top-down. The tree representations in different time windows are then encoded using Temporal GRU Encoder. Finally, we use the resulting representation of the conversation thread to calculate the probability of it being a rumor. We evaluate our model’s performance using the PHEME dataset, and instead of randomly dividing conversation threads from all events into training and test sets, we perform Leave-One-Event-Out (LOEO) cross-validation, which is closer to a realistic scenario. The results show that our proposed Temporal Tree Transformer model with Top-down integration achieves state-of-the-art classification results across multiple evaluation metrics. The results suggest that extracting temporal features from propagation leads to better generalization of model predictions.

There are numerous avenues to explore in future research. One possibility involves incorporating non-textual data, such as images and videos, to improve the representation of individual post. Additionally, assessing user credibility in the dissemination of misinformation presents a promising opportunity.

## Supporting information

S1 Table Experimental results of varying the value of k in LOEO validation for TTT - G (BU). (PDF)

S2 Table Experimental results of varying the value of k in LOEO validation for TTT - G (TD). (PDF)

## Footnotes

1 BERT-Tokenizer is retrieved from https://huggingface.co/bert-base-uncased.
