# Supplementary material for: Rumor detection on social networks based on Temporal Tree Transformer
Source: PLoS One. 2025 Apr 7;20(4):e0320333. doi: 10.1371/journal.pone.0320333 (PMC11975086; doi:10.1371/journal.pone.0320333)
Supplement: S1 Table — (PDF) [file pone.0320333.s001.pdf]

S1 Table. Experimental results of varying the value of k in LOEO validation for TTT - G (BU).

k	Event	Accuracy	Macro F1	Rumor F1	Non-rumor F1	Accuracy_Mean	Macro F1_Mean
3	CH	0.8282	0.7710	0.6567	0.8854	0.7229	0.6853
	SS	0.6920	0.6530	0.5366	0.7693		
	FG	0.7099	0.6368	0.4740	0.7997		
	OS	0.7071	0.6998	0.6528	0.7467		
	GC	0.6774	0.6658	0.6037	0.7280		
4	CH	0.8372	0.7801	0.6680	0.8921	0.7413	0.6865
	SS	0.6903	0.6424	0.5114	0.7733		
	FG	0.7653	0.6071	0.3577	0.8565		
	OS	0.7165	0.7117	0.6747	0.7487		
	GC	0.6973	0.6911	0.7348	0.6474		
5	CH	0.8297	0.7720	0.6573	0.8867	0.7460	0.7027
	SS	0.6954	0.6577	0.5441	0.7713		
	FG	0.7644	0.6484	0.4465	0.8503		
	OS	0.7211	0.7163	0.6792	0.7534		
	GC	0.7196	0.7190	0.7065	0.7316		
6	CH	0.8252	0.7749	0.6686	0.8813	0.7540	0.7123
	SS	0.7099	0.6836	0.6923	0.7748		
	FG	0.7693	0.6415	0.4275	0.8555		
	OS	0.7410	0.7394	0.7190	0.7597		
	GC	0.7246	0.7221	0.6959	0.7483		
7	CH	0.8307	0.7742	0.6613	0.8871	0.7508	0.7139
	SS	0.7329	0.7223	0.6681	0.7766		
	FG	0.7614	0.6470	0.4460	0.8479		
	OS	0.7270	0.7240	0.6953	0.7526		
	GC	0.7022	0.7022	0.7044	0.7000		
8	CH	0.8437	0.7628	0.6242	0.9013	0.7406	0.7011
	SS	0.7321	0.7173	0.6527	0.7819		
	FG	0.7455	0.6580	0.4850	0.8310		
	OS	0.6943	0.6855	0.6331	0.7380		
	GC	0.6873	0.6818	0.6400	0.7237		
9	CH	0.8357	0.7757	0.6598	0.8917	0.7531	0.7083
	SS	0.7116	0.6858	0.5957	0.7759		
	FG	0.7683	0.6112	0.3641	0.8584		
	OS	0.7503	0.7498	0.7390	0.7606		
	GC	0.6998	0.7190	0.7065	0.7398		

10	CH	0.8297	0.7515	0.6121	0.8909	0.7370	0.6948
	SS	0.6809	0.6334	0.5013	0.7654		
	FG	0.7208	0.6393	0.4679	0.8107		
	OS	0.7165	0.7127	0.6798	0.7455		
	GC	0.7370	0.7370	0.7350	0.7389		
15	CH	0.8277	0.7681	0.6505	0.8856	0.7339	0.6935
	SS	0.7065	0.6774	0.5805	0.7743		
	FG	0.7564	0.6648	0.4896	0.8401		
	OS	0.6616	0.6423	0.5593	0.7254		
	GC	0.7171	0.7150	0.7397	0.6902		
20	CH	0.8272	0.7700	0.6554	0.8847	0.7370	0.7006
	SS	0.7201	0.6970	0.6132	0.7807		
	FG	0.7535	0.6686	0.5010	0.8363		
	OS	0.6768	0.6642	0.5991	0.7292		
	GC	0.7072	0.7032	0.6685	0.7378		
