# Supplementary material for: Rumor detection on social networks based on Temporal Tree Transformer
Source: PLoS One. 2025 Apr 7;20(4):e0320333. doi: 10.1371/journal.pone.0320333 (PMC11975086; doi:10.1371/journal.pone.0320333)
Supplement: S2 Table — (PDF) [file pone.0320333.s002.pdf]

S2 Table. Experimental results of varying the value of k in LOEO validation for TTT - G (TD).

k	Event	Accuracy	Macro F1	Rumor F1	Non-rumor F1	Accuracy_Mean	Macro F1_Mean
3	CH	0.8463	0.7738	0.6458	0.9019	0.7400	0.6869
	SS	0.6819	0.6450	0.5306	0.7595		
	FG	0.7679	0.6256	0.3947	0.8564		
	OS	0.6717	0.6587	0.5922	0.7253		
	GC	0.7320	0.7314	0.7441	0.7188		
4	CH	0.8332	0.7753	0.6612	0.8894	0.7560	0.7147
	SS	0.6863	0.6534	0.5468	0.7601		
	FG	0.7467	0.6316	0.4256	0.8375		
	OS	0.7547	0.7539	0.7401	0.7677		
	GC	0.7593	0.7593	0.7628	0.7557		
5	CH	0.8312	0.7805	0.6750	0.8860	0.7347	0.6873
	SS	0.6766	0.6204	0.4743	0.7665		
	FG	0.7564	0.6328	0.4198	0.8459		
	OS	0.7048	0.6989	0.6567	0.7410		
	GC	0.7047	0.7040	0.6893	0.7187		
6	CH	0.8367	0.7535	0.6103	0.8967	0.7604	0.7129
	SS	0.7543	0.7510	0.7225	0.7795		
	FG	0.7614	0.6133	0.3740	0.8526		
	OS	0.7375	0.7374	0.7331	0.7417		
	GC	0.7122	0.7091	0.7387	0.6796		
7	CH	0.8387	0.7683	0.6407	0.8960	0.7514	0.7174
	SS	0.7253	0.7081	0.6374	0.7788		
	FG	0.7455	0.6642	0.4990	0.8295		
	OS	0.7305	0.7298	0.7166	0.7430		
	GC	0.7171	0.7166	0.7047	0.7286		
8	CH	0.8415	0.7825	0.6694	0.8955	0.7584	0.7198
	SS	0.7483	0.7433	0.7076	0.7790		
	FG	0.7752	0.6533	0.4477	0.8589		
	OS	0.7025	0.6954	0.6492	0.7416		
	GC	0.7246	0.7245	0.7286	0.7204		
9	CH	0.8317	0.7703	0.6515	0.8890	0.7376	0.7046
	SS	0.7346	0.7280	0.6855	0.7705		
	FG	0.7297	0.6674	0.5236	0.8113		
	OS	0.7445	0.7435	0.7280	0.7591		
	GC	0.6476	0.6140	0.5000	0.7280		

10	CH	0.8357	0.7691	0.6451	0.8931	0.7104	0.6545
	SS	0.7048	0.6713	0.5664	0.7762		
	FG	0.7545	0.6466	0.4513	0.8418		
	OS	0.5624	0.5013	0.3268	0.6759		
	GC	0.6948	0.6841	0.7421	0.6216		
15	CH	0.8052	0.7572	0.6493	0.8651	0.7276	0.6941
	SS	0.7082	0.6751	0.5714	0.7788		
	FG	0.7317	0.6595	0.5028	0.8163		
	OS	0.6931	0.6842	0.6311	0.7373		
	GC	0.6998	0.6943	0.7352	0.6533		
20	CH	0.7957	0.7522	0.6453	0.8560	0.7361	0.6952
	SS	0.6621	0.5947	0.4294	0.7600		
	FG	0.7762	0.6717	0.4864	0.8570		
	OS	0.7456	0.7456	0.7441	0.7471		
	GC	0.7010	0.7118	0.7225	0.7010		
